# Patterns of livestock depredation by tiger (*Panthera tigris*) and leopard (*Panthera pardus*) in and around Corbett Tiger Reserve, Uttarakhand, India

**DOI:** 10.1371/journal.pone.0195612

**Published:** 2018-05-24

**Authors:** Harendra Singh Bargali, Tanveer Ahmed

**Affiliations:** 1 The Corbett Foundation, Dhikuli, Ramnagar, District-Nainital, Uttarakhand, India; 2 Department of Wildlife Sciences, Aligarh Muslim University, Aligarh, India; Centre for Cellular and Molecular Biology, INDIA

## Abstract

India with estimated more than 2000 tigers (across 18 states) accounts for more than half of the remaining tigers across its range countries. Long-term conservation requires measures to protect the large carnivores and its prey base beyond the Protect Areas. The Corbett Tiger Reserve (CTR) and adjoining forest divisions with high density of tigers play a crucial role in conservation of tiger in Uttarakhand state as well as the Terai-Arc Landscape. However, CTR is surrounded with multiple-use forest (forest divisions), agriculture land, human habitation, townships and developmental projects. The movement of large carnivores and other wildlife through such habitats adds to the chances of human-wildlife conflict. The aim of the current study was to understand the patterns of livestock depredation by tigers and leopards in and around CTR. We examined a total of 8365 incidents of livestock depredation between 2006 and 2015 with tigers killing more livestock in a year (573.3±41.2) than leopards (263.2±9.9). Geographically, in north zone of CTR leopards were the major livestock predator (166.6±11), whereas tigers (547.7±40.1) in south zone. Examination of livestock kills indicated cows (75%) as the main victim, followed by buffaloes and other species. Analysis revealed that the livestock depredation by tigers varied significantly among seasons in south zone but not in north zone. However, such an explicit seasonal variation was not observed for leopards in north and south zone of CTR. Hotspots of livestock predation were identified around CTR. Addressing a conflict situation in a time-bound manner, timely disbursement of ex-gratia payment, involving locals at various tourism related activities and consistent rapport building initiatives are required to mitigate the human-wildlife conflict.

## Introduction

Large carnivores are declining across their distribution range [1, 2, 3].The tiger (*Panthera tigris*), the largest felid species, historically ranged in much of Asia including the regions between the Caspian and Aral Seas, South-eastern Russia and the Sunda islands [1, 2, 3]. Currently almost all but 7% of the tigers original range has been lost in last 150 years resulting in sharp decline in the tiger distribution in its historic range [4]. Its congener, leopard (*Panthera pardus)* occurring across much of Africa and Asia from the Middle East to Pacific Ocean now restricted to 25–37% of its historic range [5]. Habitat loss, fragmentation of remaining habitat, poaching for trade in body parts, prey depletion, hunting and lack of law enforcement and conflict with the people are among the major challenges to the survival of species across its range [4, 5, 6, 7, 8]. To ensure conservation of tiger, the Government of India launched “Project Tiger” in 1973 focusing primarily on protection of tiger, its prey species and habitat. Currently, with 2,226 (1945–2491) tigers distributed in 18 states, India accounts for about 60% free-ranging tigers [8]. About 70% of the tiger population exist within Protected Areas (PAs). PAs serve as sources and contiguous forests along with corridors outside PAs facilitate the dispersal of tiger and other carnivores towards sinks. Hence habitat outside PAs ensures long-term demographic and genetic variability [4, 8].

Alternatively, communities living in the vicinity of PAs suffers from limited historical rights, restrictions in traditional livelihoods and insignificant role of local communities in managing and protecting such designated areas [9, 10, 11, 12, 13]. In addition, crop damage by wild herbivore, livestock depredation and human casualties by tiger and other carnivores impose diverse and pervasive cost on local communities resulting in hostility towards conservation [14, 15, 16, 17, 18, 19, 20, 21, 22]. Therefore, a fair understanding of such issues impacting local communities living in and around PAs is fundamental in balancing conservation goals [23, 24, 25, 26, 27, 28].

The current study aims at understanding the patterns of livestock depredation by large carnivores in and around CTR. The area supports high density of tigers with 9.4 tigers/100 km^2^ in Corbett landscape and 14 tigers/100 km^2^ in Ramnagar forest division) [29]. Number of tigers has increased significantly in the landscape over the years and currently there are 215 (169–261) tigers within CTR [8]. In such a situation, conservation depends on ensuring large carnivore management outside CTR in adjoining territorial forest divisions facilitating tiger and other carnivore movement across the Terai-Arc Landscape (TAL). The TAL, identified as a landscape of global importance, is home to flagship species such as tigers, elephants and rhinos [30, 31, 32, 33]. It is expected that outcome of the study will be useful in planning future management strategies to mitigate the issue of livestock depredation which will play a crucial role in ensuring long-term conservation of large carnivores in TAL.

## Study area

**Fig 1:**
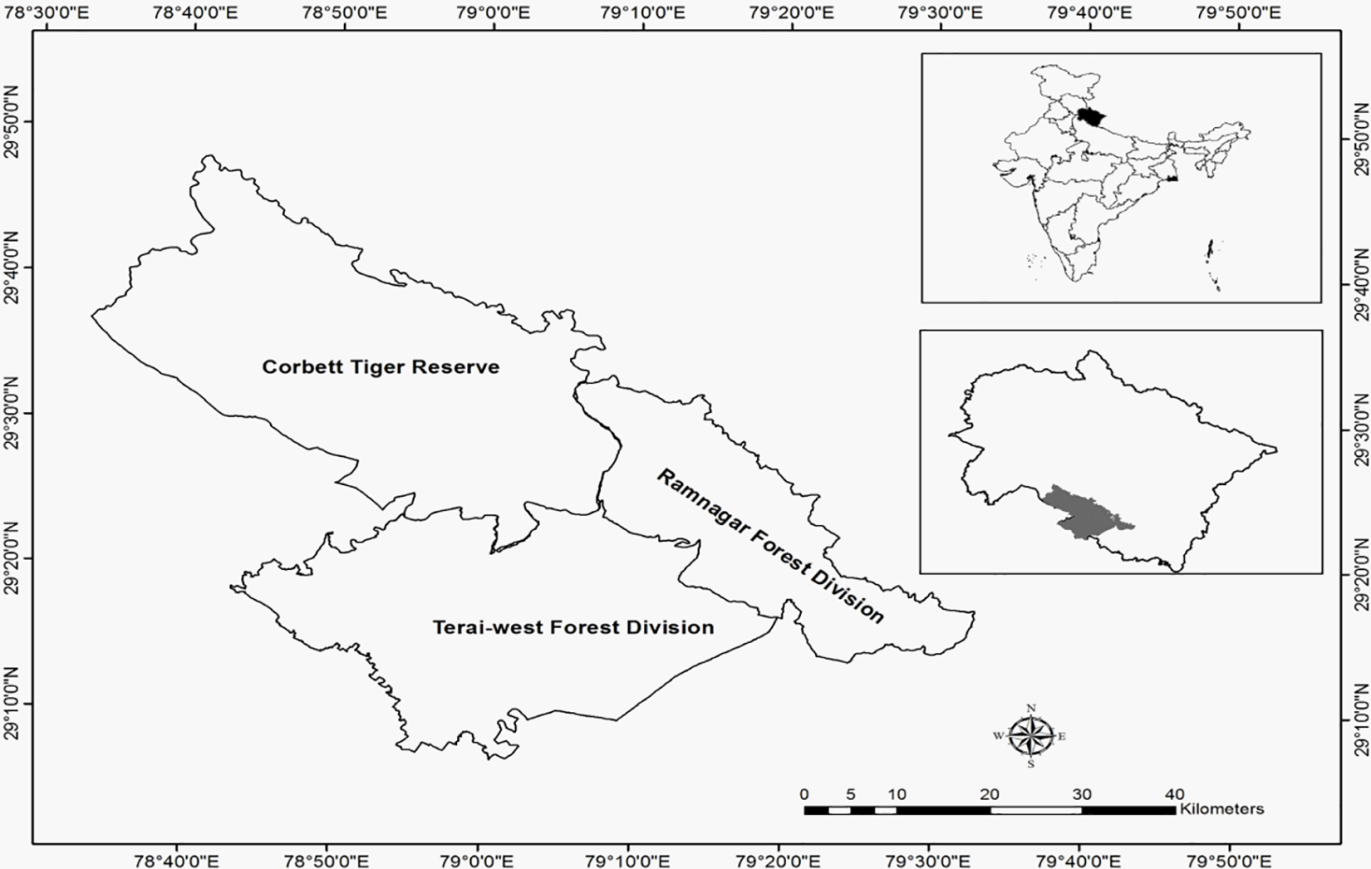
Map of the study area showing Corbett Tiger Reserve and adjoining forest divisions.

The Corbett Tiger Reserve (Latitudes 29^0^ 48’ N- 29^0^ 15’ N & Longitudes 78^0^ 39 E- 79^0^ 27’ E), encompassing an area of 1368.91 km^2^ with elevation varying between 385 and 1100 m asl, is located in Uttarakhand state, India (Fig 1). Administratively, it falls under three districts viz. Nainital and Pauri (Uttarakhand) and Bijnore (Uttar Pradesh). CTR is surrounded by Ramnagar, Almora, Terai-West, Pauri and Bijnore Forest divisions.

Geographically, it is flanked by the Shivaliks on the north and to the south by Gangetic plains. Topography of the region is characterized by hilly terrain with coarse soil and boulders in the north and fine alluvium and clay rich swamps with a shallow water table in the south. Ramganga, Palain, Mandal and Sonanadi are important rivers flowing through the CTR. The area receives an average annual rainfall of 1925mm, mostly during the southwest monsoon (June-September).

The vegetation in and around CTR is categorised as Moist Siwalik Sal Forest, Moist Terai Sal Forest, West Gangatic Moist Mixed Deciduous Forest, Khair Sissoo Forest, Northern Dry Mixed Deciduous Forest, Dry Siwalik Sal Forest, Dry Plains Sal forest, Dry Deciduous Scrub, Upper or Himalayan Chir Pine Forest, Himalayan Sub Tropical Scrub, Oak Forest (Q.incana), Western Mixed Coniferous Forest (Spruce, Blue Pin), and Plantation [34, 35]. These forests support 617 flora and 1013 faunal species including 49 mammals, 685 birds, 39 reptiles and 36 pisces [36]. CTR has high density of tiger (17.8 tigers/100 km2) along with the highest concentration of Asian elephants *Elaphus maximus* with an estimated 1035 individuals [29, 37] in 2015.

## Methodology

Incidents of livestock depredation within 5 km from the boundaries of CTR and upto 30 km within Ramnagar forest division were visited between 2006 and 2015. The information was collected under a collaborative Interim Relief Scheme conducted by The Corbett Foundation (TCF) and World Wide Fund for Nature-India. Under the scheme, in case of livestock depredation and to claim for interim relief, villagers have to inform TCF within 72 hours of the incident. The scheme intends timely disbursement of interim relief to avoid the chances of carcass poisoning and promote harmonious coexistence between local communities and large carnivores. The interim relief is an on-site immediate financial supports in addition to the ex-gratia provided by concerned forest department.

**Fig 2:**
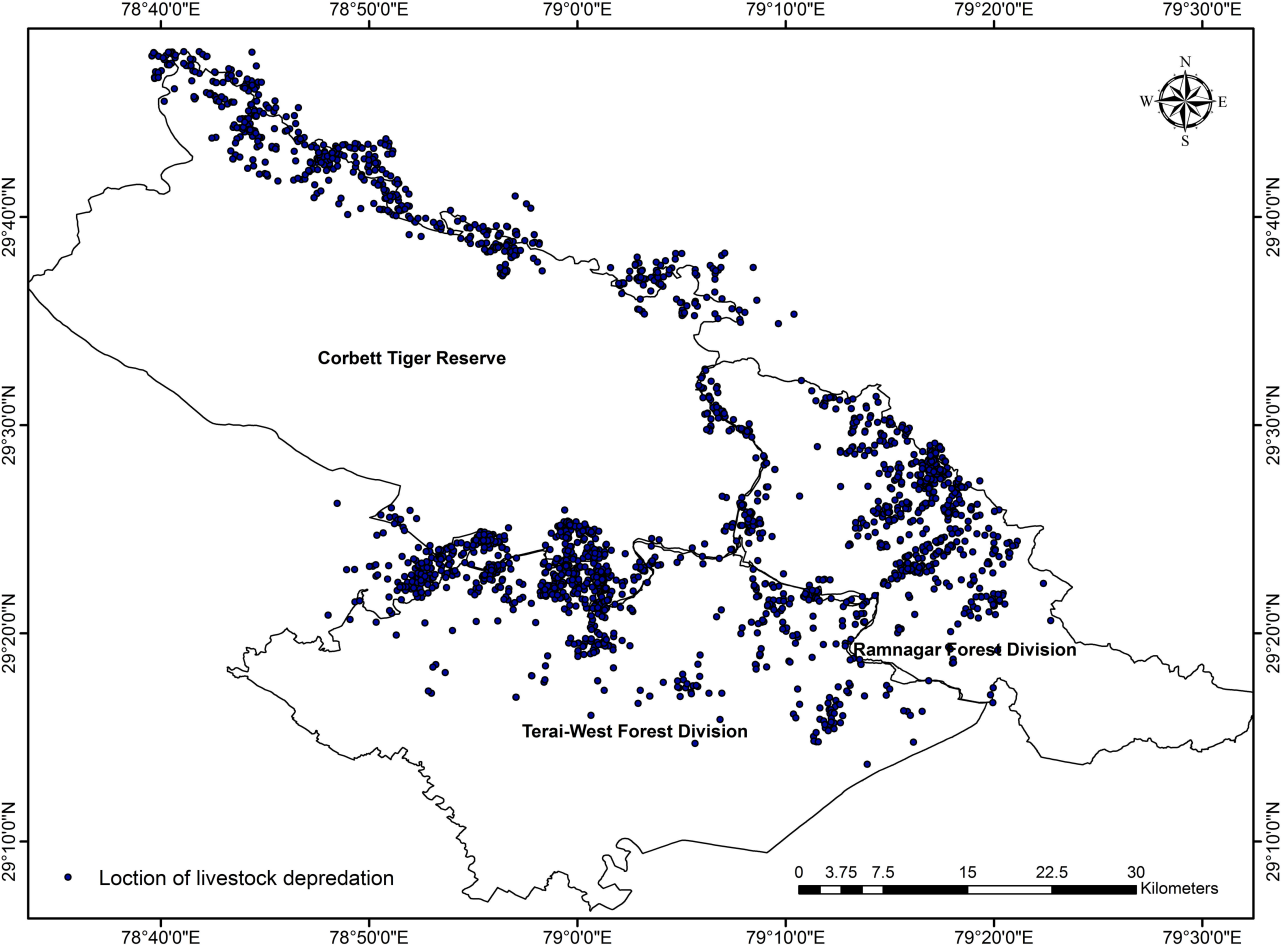
Locations of livestock depredation by the tigers and leopards in and around CTR.

Locations of livestock depredation were visited and data on species, sex, age, GPS location, season etc. were recorded. Identification of predator was based on the direct sightings at carcass, observations of villagers, indirect evidences and patterns of carcass consumption. Livestock species were grouped in three categories viz. a) cow (male, female and calf), b) buffalo (male, female and calf) and others (horse, mule and donkey). Kruskal-Wallis One Way ANOVA was used to determine the sesonal difference in livestock depredation by tigers and leopards. To determine the hotspot of conflict, we overlay a 2x2 Km grid over the study area and calculated the conflict incidents in each grid. A total of 2773 livestock depredation incidents occurred between 2012 and 2015 were used to map conflict hotspot (Fig 2). GPS locations of each of the incident prior to year 2012 were not available. We used Inverse Distance Weighted Interpolation (IDW) technique in Spatial Analyst tool of Arcmap 10.0 to determine the hotspots of conflict. IDW is a geospatial interpolation technique based on the assumption the closer the samples are from each other, the more similar would be their values.

Financial loss incurred was calculated by considering the prevaling market value of various age categories of livestock. The approximate monetary value of different-aged livestock was a) $ 38 for calf to $ 156 for female adult cow b) $ 187 for calf to $779 for female adult buffaloes c) $ 233 for calf to $ 389 for adult horse/mule (S1 Table). The values of different livestock species were converted to United States dollars at an exchange rate of USD 1: INR 64.1.

## Results

**Fig 3:**
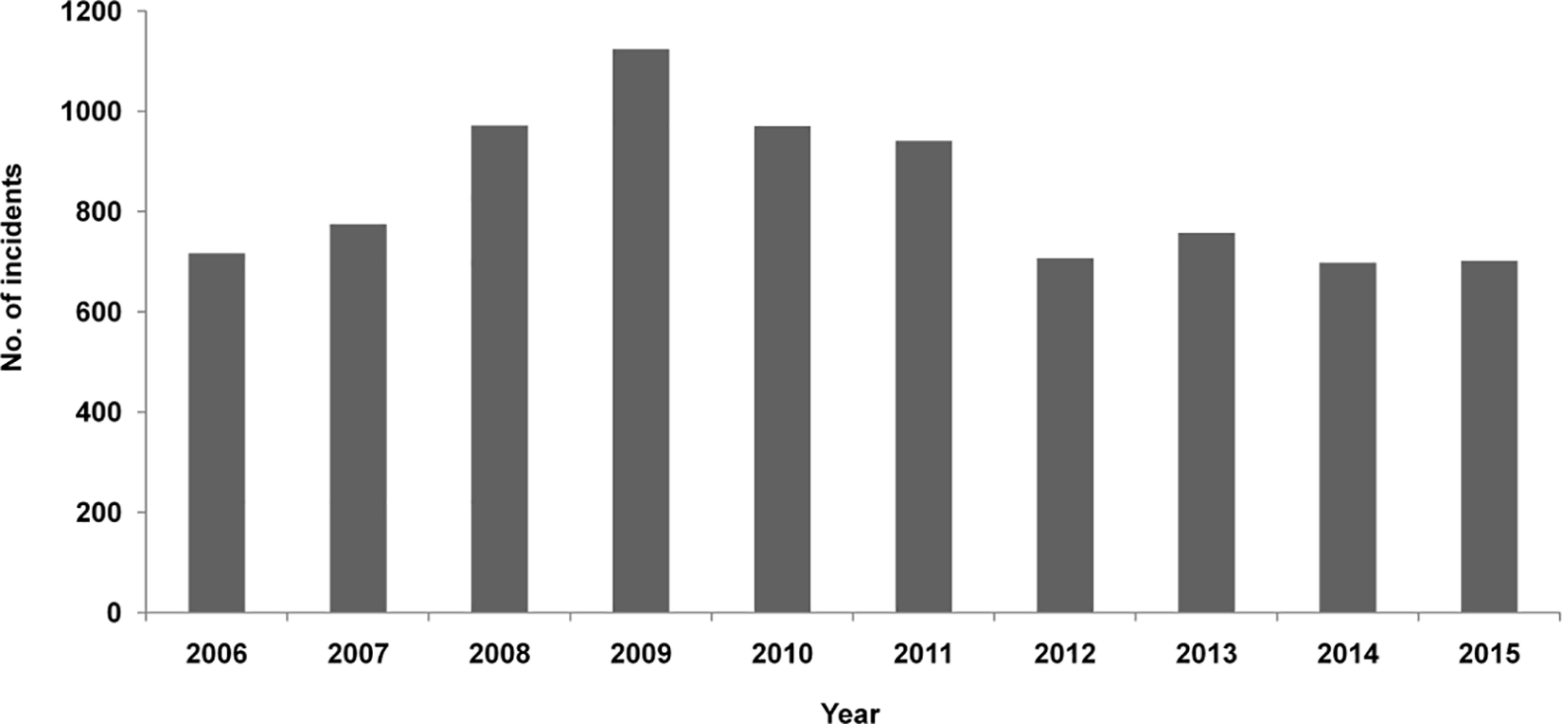
Yearly variation in livestock depredation by tigers and leopards during 2006–2015.

A total of 8,365 incidents of livestock depredation were recorded from 356 villages and Gujjar settlements (a previously nomadic, now sedentary forest dwelling tribal-community) between 2006–2015. Most of the incidents of livestock depredation were by tigers (573.3±41.2) than leopards (263.2±9.9). Maximum incidents of livestock depredation (n = 1124) were recorded in 2009 and minimum (n = 698) in 2014 (Fig 3, S2 Table).

**Fig 4:**
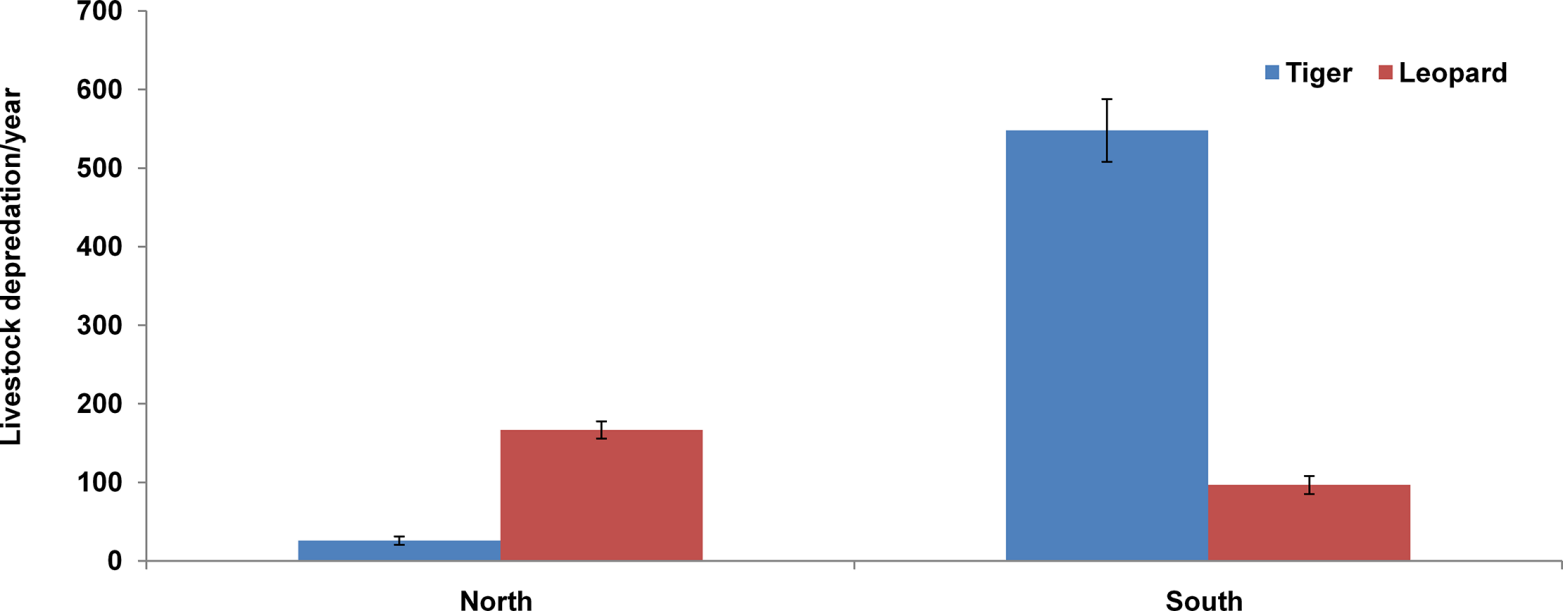
Livestock depredation by tigers and leopards in north and south zone of CTR during 2006–2015.

**Fig 5:**
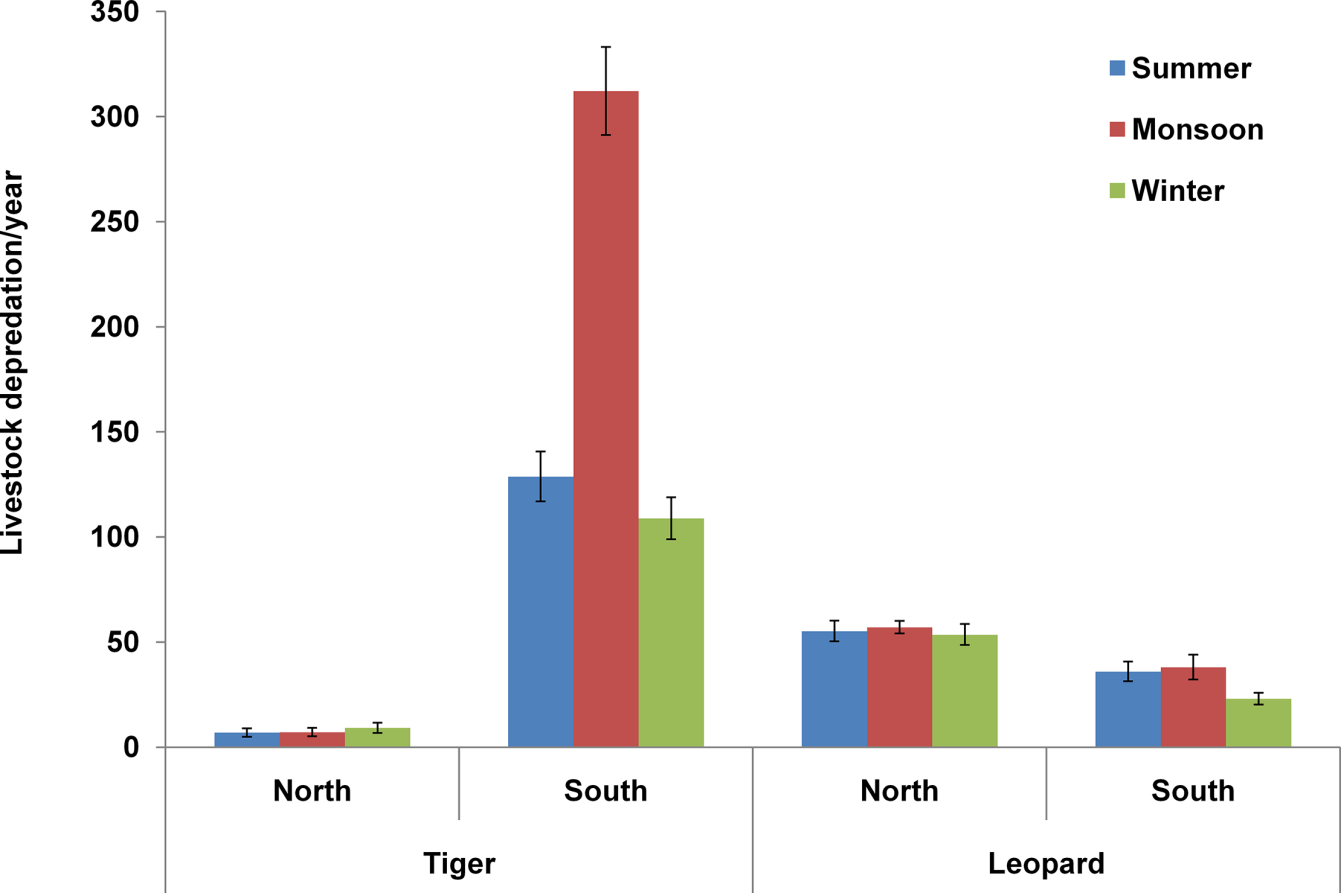
Seasonal variation in livestock depredation by tigers and leopards in north and south zone of CTR during 2006–2015.

Incidents of livestock depredation were recorded more in South zone (644.3±41.5) than North zone (192.2±11.9) of CTR. Livestock depredation incidents around CTR indicated that leopards were the main predator in north zone (166.6±11) and tigers (547.7±40.1) in south zone (Fig 4, S3 Table, S4 Table). In south zone, a comparision of livestock depredaton incidents among seasons revealed that depredation by tigers was comperatively more during monsoon season (312.20±20.90) than summer (128.80±11.89) and winter (108.90±9.98). There was significant difference in livestock depredation by tigers among various seasons (KW = 19.9 df = 2 P < .05), however, such marked seasonal difference was not observed for leopards (Fig 5). Seasonal livestock depredation by both the predators was not statistically significant in north zone.

**Fig 6:**
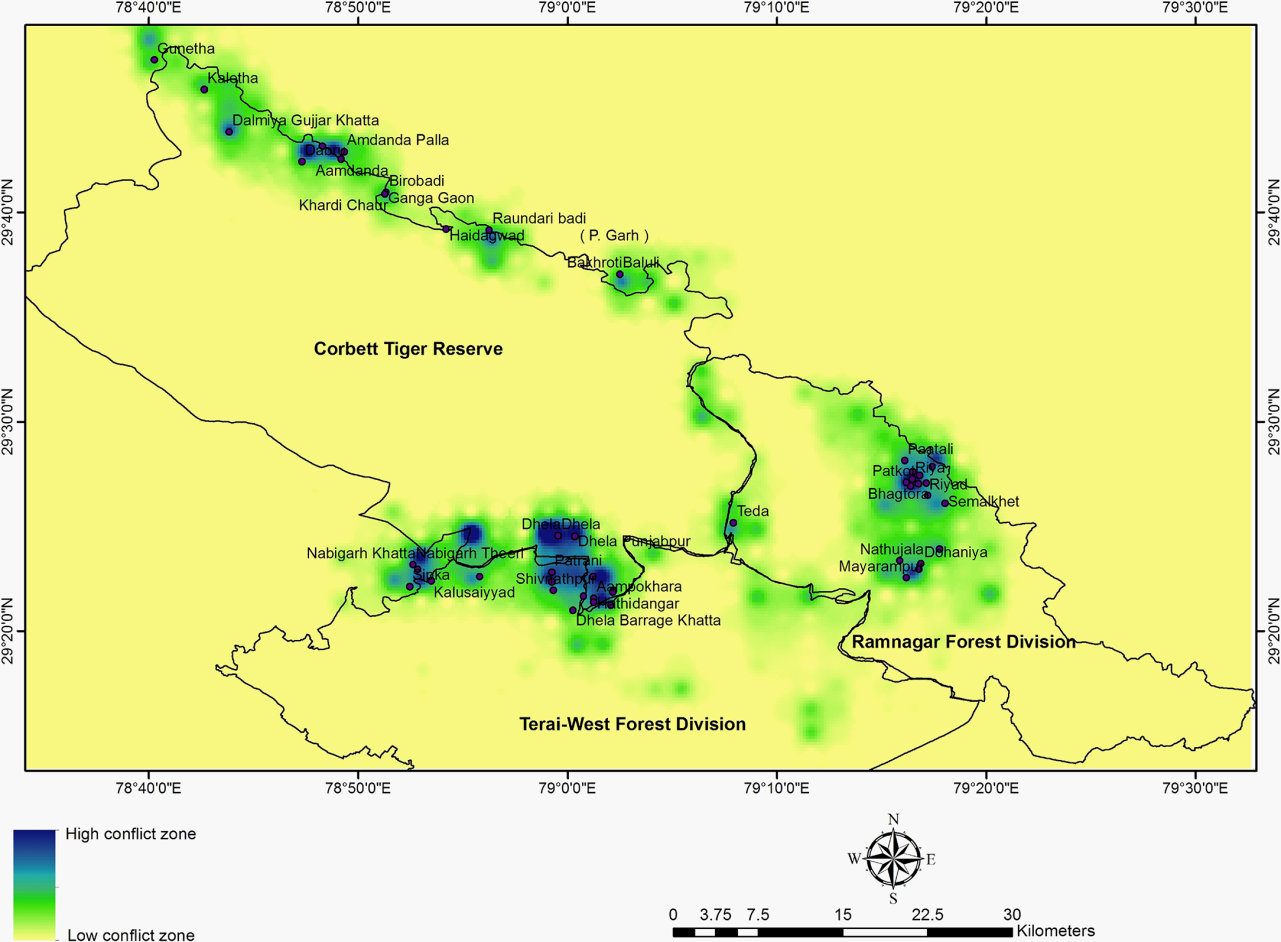
Hotspots of livestock depredation in Corbett landscape with yellow depicting low conflict and blue high-conflict areas.

Overall, cow (75.0%) was the major victim of tigers and leopards followed by buffalo (24.5%) and other species (0.5%). Pattern of livestock depredation differed in tigers and leopards. Tigers mostly prey upon cow (66%) followed by buffalo (33.5%) and others (0.5%), whereas, leopard predation was mainly on cows (95.0%). Our analysis indicated conflict hotspot along the northern and south eastern boundary of CTR. Parts of Nainital, Almora, Pauri, and Bijnore administrative districts were found to be affected due to livestock depredation by tigers and leopards. Major conflict hotspots were also observed at the north central and central part of Ramnagar Forest Division (Fig 6). The area around Gunetha, Kaletha, Dalmiya Gujjar Khatta, Dabru, Ganga Gaon, Raundary badi Amdanda, Amdanda palla, Bhakroti, Baluli (Northern boundary of CTR), Belghatti Khatta, Bhawanipur, Kalusayyad, Sipka, Nabigarh, Theeri, Dhela, Ampokhra, Phanto, Khulbey (South eastern boundary of CTR), Pathkot, Amotha, Amtoli, Riyar, Simal khet, Nathujhala, Dohaniya and Mayarampur (RFD) fall under conflict hotspots.

Economic loss to local community in terms of livestock depredation by tigers and leopards was calculated approximately INR 1,57,19,200 or USD 245152.84 per year between 2006–2015.

## Discussion

The estimated number of tigers in India has increased from 1,411(1,165 to 1,657) in year 2006 to 1,706 (1,520 to 1,909) in 2010 and 2226 (1945 to 2491) in 2014 [8]. In Uttarakhand, tigers population has increased from 178 (161–195) in 2006, to 227 (199–256) in 2010 and 340 (299–381) in 2014. CTR alone supports more than 200 tigers in Uttarakhand.

Tigers and leopards were the major predators in and around CTR. Predation on domestic livestock by the two predators has been observed in several studies [38, 39, 40, 41, 42]. In our study, tiger depredated from cow to buffalo whereas, leopard largely concentrated on cow. Tiger diet is biogeographically diverse with preference varying from large prey to medium sized prey species [43, 38, 44, 45]. Prey selection in large carnivore species is based on body size [46], tiger prefer larger livestock whereas, leopard prefer small sized livestock prey [46].

Overall, incidents of livestock depredation were recorded more in south zone than north zone. This might be due to high density of tigers in south zone. Besides the incidents of livestock depredation were also recorded beyond 5 km from the boundaries of CTR in Ramnagar forest division. Tiger was found to be the major predator in south zone while leopard in north zone. This could be related to the topography of the CTR. The northern part of CTR is more rugged and hilly in comparison to south zone where leopard population can sustain well. Tiger predilection for medium size prey species may disturb other carnivores such as leopard in the same habitat [43, 46, 47] which might be the reasons behind high number of livestock depredation by tigers in south zone.

More incidents of livestock depredation by tiger during monsoon season in south zone could be ascribed to the fact that during monsoon season it becomes difficult for the tiger to catch the natural prey owing to increased vegetation cover and availability of water in the forest. During other season, it is comparatively easy to prey along limited water sources. Such a seasonal trend was not observed for leopards in north zone. This could be due to undulating topography and availability of water for prey species in several streams across the year.

There were more hotspots at the north zone, whereas, in case of south zone most of the villages under severe conflict zone fall within a larger hotspot. Similarly, hotspot in RFD also indicate use of the forested habitats of the division as corridor by both tigers and leopards in the landscape.

Considering the increasing number of tigers within the study area and human dominated habitats outside CTR where the communities incur significant economic loss, conservation of tigers and leopards will depend on support from local communities. This can be ensured by addressing the issue of human-wildlife conflict in an effective manner. In such a scenario, long term conservation of wildlife requires collaborative and comprehensive planning benefitting both local communities and wildlife. The Interim Relief Scheme has proved as one of the successful interventions in preventing likelihood of retaliatory killing in areas covered under scheme for more than a decade [48, 49]. Need for more such scheme addressing human-wildlife conflict and securing wildlife habitats outside PAs are required to ensure long-term conservation of large carnivores in TAL.

### Recommendations

Biodiversity conservation in human-dominated landscapes is a challenging task. In India, about 5 million people live inside nature reserves, and a further 147 million depend on resources provided by these reserves [50]. Outside protected areas local communities are resource dependent, politically linked and having diverse viewpoints [43, 51, 52, 53, 54, 55]. The local communities in the Corbett landscape incur economic loss through livestock depredation and reap economic benefits through wildlife tourism along with fuelwood and fodder. It is important to involve local communities in seeking situations to balance these costs and benefits [56]. However, a practical reconciliation has yet to be achieved [57].

Hence it is strongly recommended that forest divisions outside PAs should have adequate funds to ensure timely disbursement of ex-gratia payments. Secondly, CTR generates revenue in millions of USD yearly through tourism [58]. A part of such revenue is suggested to be mobilised to meet the demand of employment, education, primary health check-up facilities and timely disbursement of ex-gratia payments [59]. Thirdly, Gujjars living within the buffer zone of CTR and adjoining forest divisions have expressed their strong desire to be relocated from these forests [60]. It is suggested that relocation of Gujjars and other communities willing to relocate should be considered on priority. In conclusion, collaborative policy is needed to manage the habitats outside CTR to ensure long-term conservation of tigers and leopards in and around CTR.

## Supporting information

S1 Table Market value of livestock and average economic loss. (XLSX)

S2 Table Livestock depredation by different predator species. (XLSX)

S3 Table Livestock depredation at North and South zone of CTR. (XLSX)

S4 Table Livestock depredation by different predators in various zones of CTR. (XLSX)
